# The Use of Perceval Valves in Older Patients: A Systematic Review

**DOI:** 10.31083/RCM39463

**Published:** 2025-07-28

**Authors:** Ivo Deblier, Dina De Bock, Inez Rodrigus, Wilhelm Mistiaen

**Affiliations:** ^1^Department of Cardiovascular Surgery, ZAS Middelheim Hospital, 2020 Antwerp, Belgium; ^2^Department of Cardiovascular Surgery, UZA – University Hospital Antwerp, 2650 Edegem, Belgium; ^3^Faculty of Medicine & Health Sciences, University of Antwerp, 2610 Antwerp, Belgium

**Keywords:** aortic valve replacement, Perceval, permanent pacemaker implantation

## Abstract

**Background:**

The Perceval device is a sutureless, rapid-deployment valve designed to shorten aortic cross-clamp (ACC) and cardiopulmonary bypass (CPB) times, with the aim of improving postoperative outcomes in older, high-risk patients.

**Methods:**

A systematic review was conducted for full articles published between 2020 and 2024, comparing the Perceval valve with conventionally sutured valves, with a focus on preoperative and operative data, as well as postoperative outcomes. Single-arm series were retained for the same purpose. Articles with at least 100 valves were included.

**Results:**

A total of six propensity score-matched series and four randomized controlled trials were identified after removing articles with data from the same patient population. Consequently, age and risk scores were comparable. The use of a minimally invasive approach and the association of other procedures, such as coronary artery bypass grafting (CABG), varied depending on the research design. Adverse postoperative events were comparable for both valve types, except for the development of conduction defects, which required the implantation of a permanent pacemaker (PPM). The initial PPM implantation rate was higher for the Perceval valve, as shown in 5 of the 14 comparative series; however, this rate decreased after the adaptation of surgical techniques. A meta-analysis showed that the CPB and ACC times were significantly shorter using the Perceval valve, at 14.9 (8.2–21.5) minutes and 16.6 (12.1–21.2) minutes, respectively. Platelet counts after implantation were lower with no clinical consequences, and the hemodynamic performance of the Perceval device was acceptable and stable over time. The survival and durability of the Perceval valve were also acceptable, with a reoperation rate of 1% at the 5-year follow-up.

**Conclusions:**

The Perceval valve appears to be a suitable alternative for older, high-risk patients undergoing aortic valve replacement. Notably, the Perceval valve is associated with shorter surgical times and could facilitate the advantage of minimally invasive surgery. The need for postoperative PPM implantation remains an issue.

## 1. Introduction

Symptomatic aortic valve disease is the second most prevalent structural heart disease, and is highly lethal if left untreated [1, 2]. Aortic valve replacement through surgical means has been the standard treatment for decades, aiming to prolong life and relieve symptoms. Moreover, surgical aortic valve replacement (SAVR) can still be considered a good option for patients between 75 and 79 years to treat symptomatic aortic valve disease [3]. However, patients aged 75 years and older or with a Euroscore of at least 8% are considered unsuitable to undergo SAVR because of the increased risk related to age and possible comorbid conditions [4]. Meanwhile, transcatheter aortic valve implantation (TAVI), which has become an alternative to SAVR, has profoundly altered the approach to aortic valve disease since TAVI does not involve removing the native valve and is not without complications [5]. Furthermore, a sutureless bovine pericardial prosthesis (Sorin Perceval) can be a good surgical option in this population. The Sorin Perceval device is derived from a proven stented valve design and has been successfully used in patients since its first implant in 2007. The tissue is mounted in a nitinol stent, which can be deployed through the opened aorta, after removal of the diseased valve [5, 6]. The implantation through the second intercostal space proved feasible in a cadaveric study: decalcification, sizing, and deployment of the nitinol stent could be performed, resulting in a stable and reproducible outcome [6]. Initially, the implantation was performed through a median sternotomy [7, 8]; however, the Perceval valve can be implanted through less invasive approaches due to its rapid deployment [9, 10, 11, 12]. More recently, access through right anterior mini-thoracotomy (RAMT) has been successfully applied [10, 13]. Additionally, a low-pressure balloon dilatation was used to optimize the sealing of the annulus [8, 10, 14]. Thus, major advantages include shorter cardiopulmonary bypass (CPB) and aortic cross-clamp (ACC) times [5], with respective durations of 60 and 30 minutes [7, 8, 9, 12] or less [11] noted in early series. The shorter surgical times reduces postoperative mortality and morbidity in patients with left ventricular dysfunction or undergoing complex procedures [5]. A low hospital mortality rate of 2.4% and even lower in small-scaled patient series, where the mean age was high, indicated its safety [5, 8, 10, 12, 15]. At the same time, the early peak and mean gradient of these valves were acceptable [5, 11] and remained stable during the 1-year postoperative period [5, 12]. Migration, dislodgement, paravalvular and central valvular leaks were uncommon, and the procedure was considered feasible [5, 7, 8, 11, 15] and safe, especially in patients with severely calcified roots [8]. The need for reoperation due to paravalvular leak resulting from mispositioning was 4% [9, 13]. An asymmetrically dilated root in patients with a bicuspid aortic valve was considered a contraindication [12]. However, the long-term durability of the Perceval valve remains to be investigated [5]. The first large series, investigating the Perceval valve comprising 208 high-risk patients [9], was published in 2012, with the first reports on the increased need for permanent pacemaker (PPM) implantation [15, 16]. A 4.2% rate for postoperative need of PPM implantation was observed in another larger early series [10]. The need for PPM after SAVR is a serious event since it reduces postoperative survival [17]. However, this need has diminished over time, possibly because of improvements in surgical techniques. This review aims to assess the outcomes following Perceval valve implantation.

## 2. Literature Review

### 2.1 Search Methods

**Fig. 1.**
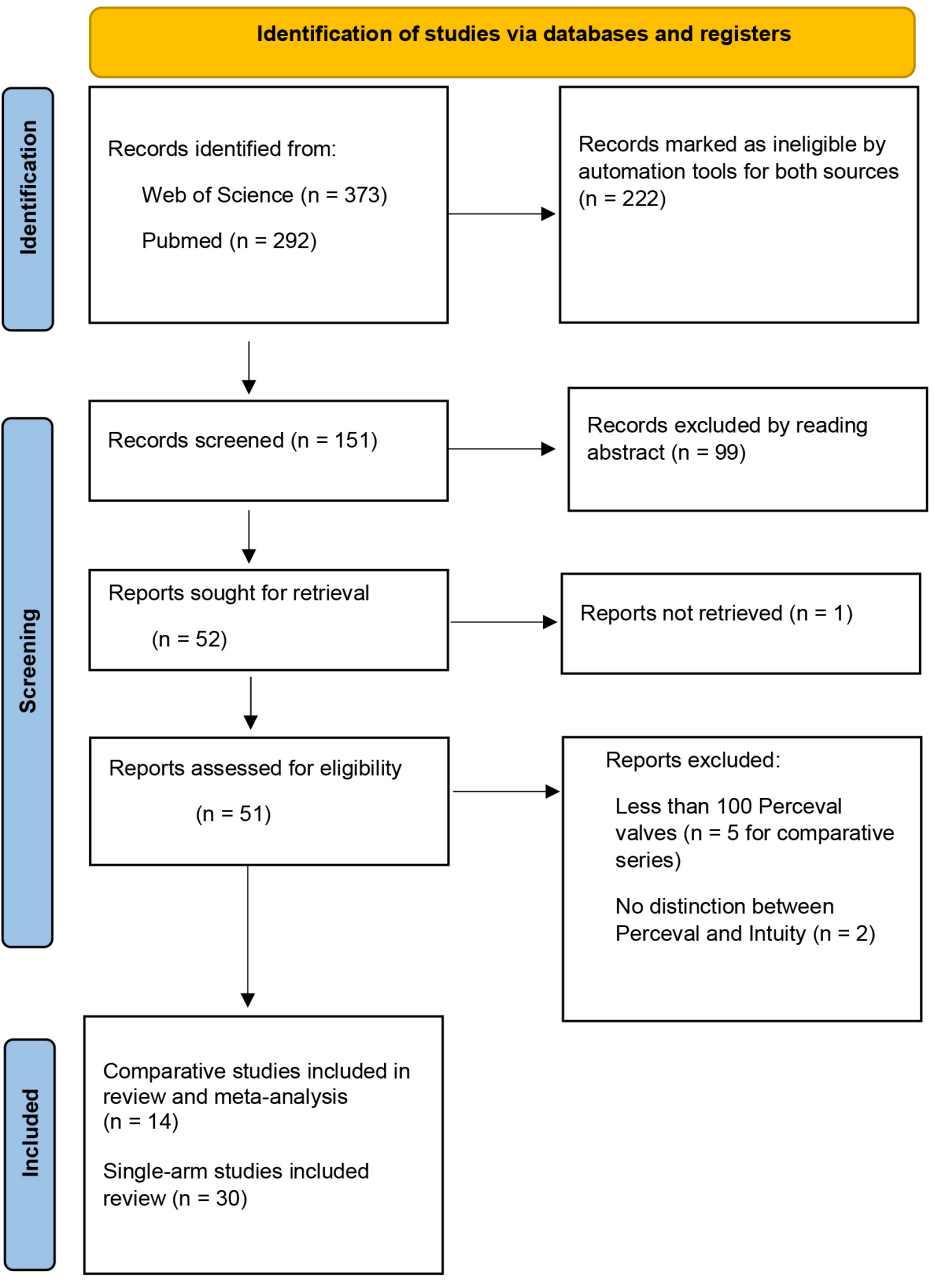
**Search strategy**.

The following search terms were used in the Web of Science and PubMed databases: “Aortic valve replacement AND Perceval”, “aortic valve replacement AND sutureless”; “aortic valve replacement AND rapid deployment NOT Intuity”, from 1/1/2020 to 31/12/2024. The exclusion criteria were case reports, case series, reviews, editorials, letters, studies without a Perceval valve implant, or grouped as sutureless and rapid deployment without specification; comparative series with TAVI; studies not primarily about valve replacement; technical descriptions; *in vitro* studies. Additionally, series with fewer than 100 Perceval valves were excluded. Following the investigation of these search terms, 373 items were identified for both sources. After excluding non-full research articles by automation, 151 manuscripts remained. Another 99 manuscripts were excluded after reading the abstract, and, if necessary, consulting the tables of the full articles (Fig. 1). Series, comparing the Perceval valve with an Intuity valve, were treated as the single-arm series for the Perceval valves, if the data concerning the Perceval valve could clearly be distinguished from those derived from the Intuity valve. A meta-analysis was performed using SPSS (version 29.0.1, IBM, Armonk, NY, USA), employing the random-effects model, with the calculation of I^2^ for inhomogeneity included to quantify the proportion of variance in effect sizes. Only one article derived from the international Perceval Sutureless Implant vs. Standard Aortic Valve Replacement (PERSIST-AVR) randomized controlled trial was included in the meta-analysis to avoid distortion of the results. All authors participated in assessing the articles obtained during the process.

### 2.2 Results

#### 2.2.1 Quality Assessment of Included Series

Table 1 (Ref. [18, 19, 20, 21, 22, 23, 24, 25, 26, 27, 28, 29, 30, 31]) shows the results of the Newcastle–Ottawa scale for quality assessment across eight aspects of the comparative series. (1) All comparative series included patients with symptomatic aortic valve disease, for whom surgical treatment was indicated and could be considered as representative. The exposed cohort consisted of individuals who received the Perceval device. (2) The so-called non-exposed cohort comprised patients who received a conventionally sutured valve. These patients were drawn from the same population. (3) The ascertainment of exposure was guaranteed by consulting the surgical records. (4) Outcomes of interest, such as mortality, postoperative stroke, postoperative bleeding, paravalvular leak, etc., were not present at the start of the study. Where necessary, patients with this outcome (such as conduction defects or PPM) were excluded from the analysis. In several reports, the outcome of interest was the change in parameters such as the mean and peak transvalvular gradient or platelet count [22, 31]. (5) Comparability was guaranteed by the study design, such as a randomized controlled trial [19, 20, 25, 26] or propensity score matching [23, 24, 27, 28, 29, 30]. In other series, patient data were derived from institutional databases, which had a clear start and endpoint for inclusion and clear exclusion criteria [18, 21]. The inclusion was explicitly consecutive [22, 31]. (6) The outcome was assessed by record linkage, echocardiography, electrocardiography, and blood exams. (7) The follow-up was adequate with respect to 30-day outcomes. Long and mid-term outcomes were determined in 6 series [19, 20, 23, 24, 30]. (8) Completeness of the follow-up was assumed for all series, since in some series, long-term outcomes were not part of the intended analyses. Overall, these criteria ensure that all these studies are of sufficiently high quality.

**Table 1.** **Newcastle–Ottawa scale for cohort studies**.

Author *et al*. [reference]	1. Exposed cohort	2. Non-exp cohort	3. Ascert. of expos	4. O.O.I not present at start	5. Comparab.	6. Outcome assesm.	7. Adequacy length of FU	8. Compl. of FU	Sum the points
Aljalloud *et al*. 2023 [18]	+	+	+	+	+	+	-	+	7
Fischlein *et al*. 2021 [19]	+	+	+	+	++	+	+	+	8
Fischlein *et al*. 2022 [20]	+	+	+	+	++	+	+	+	8
Hernandez-Vaquero *et al*. 2020 [21]	+	+	+	+	+	+	-	+	7
Jayet *et al*. 2024 [22]	+	+	+	+	+	+	-	+	7
Kaitovic *et al*. 2023 [23]	+	+	+	+	++	+	+	+	9
Kapadia *et al*. 2024 [24]	+	+	+	+	++	+	+	+	9
Lorusso *et al*. 2021 [25]	+	+	+	+	++	+	-	+	8
Lorusso *et al*. 2022 [26]	+	+	+	+	++	+	-	+	8
Paparella *et al*. 2021 [27]	+	+	+	+	++	+	-	+	8
Robich *et al*. 2023 [28]	+	+	+	+	++	+	-	+	8
Santarpino *et al*. 2022 [29]	+	+	+	+	++	+	-	+	8
White *et al*. 2022 [30]	+	+	+	+	++	+	+	+	9
Zientara *et al*. 2024 [31]	+	+	+	+	+	+	-	+	8

Ascert. of expos, ascertainment of exposure; assesm., assessment; comparab., comparability; compl., completeness; FU, follow-up; O.O.I, outcome of interest; “-” item absent, “+” present, “++” comparability for most important factor and for any secondary factor.

**Fig. 2.**
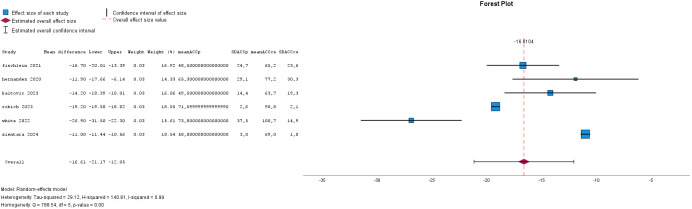
**Forest plot for aortic cross-clamp time**.

**Fig. 3.**
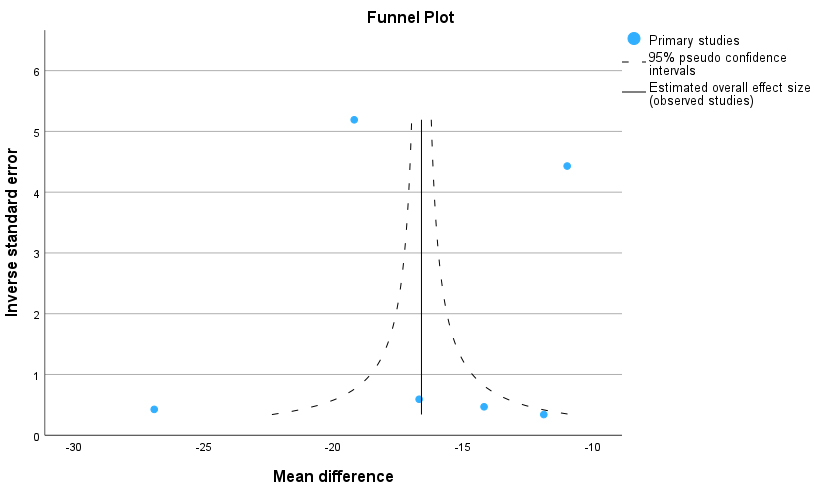
**Funnel plot for aortic cross-clamp time**.

**Fig. 4.**
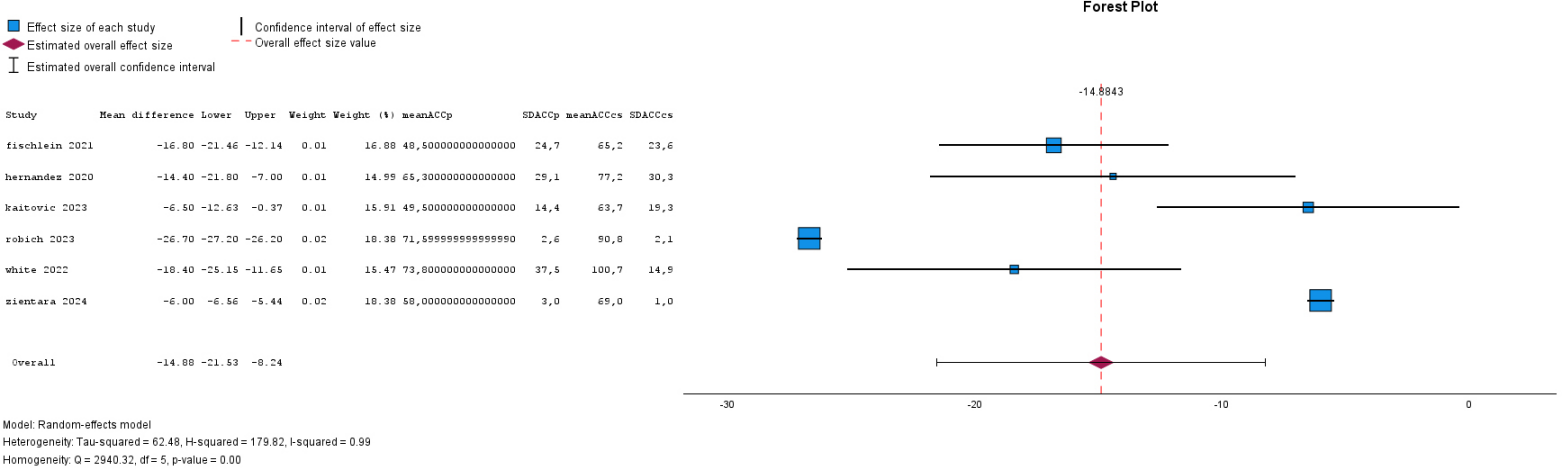
**Forest plot for cardiopulmonary bypass time**.

**Fig. 5.**
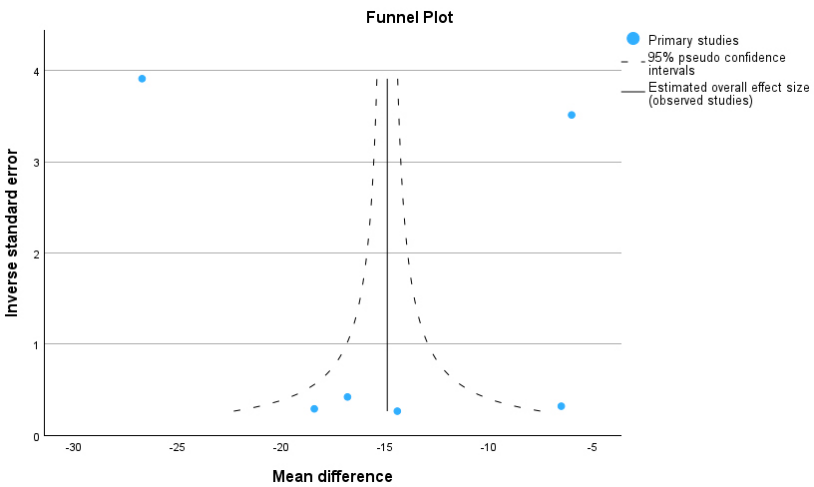
**Funnel plot for cardiopulmonary bypass time**.

In the published data (Table 2; Ref. [18, 19, 20, 21, 22, 23, 24, 25, 26, 27, 28, 29, 30, 31]) from the comparative series before propensity score matching (PSM), patients receiving the Perceval device had a mean age 3 to 4 years higher compared to those receiving conventionally sutured valves [24, 27, 28, 29]. In one series, the mean age difference was almost 10 years [31]. There were no major differences in the Euroscore before matching [24, 27]. In one series, this parameter was even lower for patients with a Perceval device [29]. Cardiac comorbid conditions varied among the included series. Although coronary artery disease was less prevalent in one Perceval group [27], in another series, there was a higher presence of prior percutaneous coronary intervention (PCI) as well as a need for associated coronary artery bypass grafting (CABG) [28]. Atrial fibrillation was more present in one Perceval group [27]. Non-cardiac comorbid conditions, such as diabetes, were lower in one Perceval group [27], while renal [28] and pulmonary [29] dysfunction were higher. Table 2 shows the four randomized controlled trials (RCTs) [19, 20, 25, 26] derived from the same patient source with very similar ages and Euroscores. For one of these series, a subdivision was made between full sternotomy and minimally invasive approach [20]. The other series [23, 24, 27, 28, 29, 30] were based on a PSM analysis, which was employed to reduce the risk of bias. The data are presented as the mean ± standard deviation or as the median with interquartile range. In these series, age and Euroscore II were also similar, which makes the inclusion of these parameters in the meta-analysis futile. The ACC and CPB times were almost universally shortened when using the Perceval valve. The meta-analysis showed a decrease in the ACC time (95% confidence interval) of 16.6 (12.1–21.2) minutes. These data are presented in Figs. 2,3. The CPB time was 14.9 (8.2–21.5) minutes, as shown in Figs. 4,5—both differences were significant. Expectedly, these surgical times increased in series with combined operations [24], but the advantage for the Perceval valve was maintained. A smaller increase in these surgical times was also observed through the employment of a minimally invasive approach, while retaining the advantage of the Perceval valve [20].

**Table 2.** **Preoperative and operative data in the comparative series**.

Author *et al*. [reference]	Valve	N	Age (y)	ES-II (%)	Full sternot.	Conc. proced.	ACC (min)	CPB (min)
Aljalloud *et al*. 2023 [18]	P	119	76.3 ± 5.6	Low ^B^	-	-	62.5 (51.0–83.0)*	74.5 (53.3–103.0) ^B^
	CS	83	73.5 ± 6.3	Low	-	-	98.0 (79.0–126)	111.3 (81.0–149.2)
Fischlein *et al*. 2021 [19]	P	507	75.4 ± 5.6	2.1 ± 1.8	49.6	30.0	48.5 ± 24.7***	71.0 ± 34.1***
	CS	412	75.0 ± 6.1	2.0 ± 1.4	47.3	28.9	65.2 ± 23.6	87.8 ± 33.9
Fischlein *et al*. 2022 [20]	P	191	75.4 ± 5.7	1.9 ± 1.6	0	-	42.1*** (fig)	63.8*** (fig)
	CS	185	74.0 ± 6.7	1.5 ± 1.8	0	-	60.4	80.6
	P	94	72.5 ± 6.0	2.0 ± 1.4	100	-	38.7***	55.5***
	CS	108	75.0 ± 5.8	1.9 ± 1.6	100	-	55.5	73.9
Hernandez-Vaquero *et al*. 2020 [21]	P	140	78.2 ± 6.4*	4.5 ± 4.6	-	10 (est)	65.3 ± 29.1***	81.3±4.9***
	CS	409	76.7 ± 5.8	5.4 ± 5.8	-	20 (est)	77.2 ± 30.3	95.7 ± 39.7
Jayet *et al*. 2024 [22]	P ^S^	103	72.8 ± 1.2	2.0 (1.3–3.9)	83	45	44 (29–63)***	58 (41–93)**
	CS	53	73.9 ± 0.8	1.9 (1.2–3.9)	98	42	63 (47–78)	75 (58–105)
Kaitovic *et al*. 2023 [23]	P	101	72.1 ± 5.6	2.3 ± 1.7	13.9**	-	49.5 ± 14.4***	83.0 ± 28.9*
	CS	238	72.8 ± 5.3	2.6 ± 3.9	66.8	-	63.7 ± 19.3	89.5 ± 25.2
Kapadia *et al*. 2024 [24]	P	54	73.7 ± 6.7	2.0 (1.5–3.0)	72	0	52 (43–63)**	82 (67–96)**
	CS	48	74.0 ± 6.4	2.0 (1.3–3.3)	95	0	70 (58–82)	91 (79–103)
	P	59				100	74 (62–91)**	110 (80–133)**
	CS	43				100	107 (92–123)	147 (118–167)
Lorusso *et al*. 2021 [25]	P	407	75.4 ± 5.6	2.2 ± 1.8	49.6	24.3	46.9 ± 20.5***	68.5 ± 27.7***
	CS	412	75.0 ± 6.1	2.0 ± 1.4	52.7	22.3	63.9 ± 22.2	85.9 ± 31.7
Lorusso *et al*. 2022 [26]	P	450	75.5 ± 5.7	2.2 ± 1.9	49.7	30.2	-	-
	CS	446	75.0 ± 6.2	2.0 ± 1.4	52.9	28.5	-	-
Paparella *et al*. 2021 [27]	P	430	77 (72–82)	1.8 (1.3–3.1)	0	-	65 (54–84)	48 (40–62)***
	CS	860	77 (72–81)	1.7 (1.2–2.9)	0	-	78 (61–91)	63 (48–74)
Robich *et al*. 2023 [28]	P	234	70.2 ± 0.64 ^B^	-	75.6*	51.1	71.6 ± 2.6***	98.9 ± 3.3***
	CS	370	69.5 ± 0.52	-	91.7	50.8	90.8 ± 2.1	125.6 ± 2.9
Santarpino *et al*. 2022 [29]	P	206	78*	2.3*	-	16	48**	65 ^B^
	CS	206	79	2.8	-	17	58	73
White *et al*. 2022 [30]	P, P ^S^	195	72.4 ± 9.5	-	-	31.9***	73.8 ± 37.5***	108.3 ± 56.4*
	CS	295	72.4 ± 9.9	-	-	28.8	100.7 ± 14.9	126.7 ± 17.8
Zientara *et al*. 2024 [31]	P	100	72 ± 1***	-	-	0	58 ± 3***	87 ± 3*
	CS	219	68 ± 1	-	-	0	69 ± 1	93 ± 2

ACC, aortic cross-clamp time; CPB, cardiopulmonary bypass time; Conc. proced., concomitant procedure; CS, conventionally sutured; ES, Euroscore; est, estimated; fig, data derived from figures in the included manuscript; N, number; P, Perceval; P ^S^, Perceval S; sternot., sternotomy; *, *p* < 0.05; **, *p* < 0.01; ***, *p* < 0.001; ^B^, borderline significant.

#### 2.2.2 Meta-Analysis for Aortic Cross-Clamp Time and Cardiopulmonary Bypass Time

The meta-analysis shows the forest plot for aortic cross-clamp time and cardiopulmonary bypass time in six series. In total 1277 patients for the Perceval group and 1943 patients for the conventionally sutured group were included. The heterogeneity (I^2^) was 0.99 and 3 studies were outside the funnel plot, indicating some publication bias. A reduction of 16.6 (12.1–21.2) minutes for aortic cross-clamp time and of 14.9 (8.2–21.5) minutes for cardiopulmonary bypass time was significant.

#### 2.2.3 Series Comparing Perceval With Conventionally Sutured Valves

The short-term outcomes are presented in Table 3 (Ref. [18, 19, 20, 21, 22, 23, 24, 25, 26, 27, 28, 29, 30, 31]). No significant difference was identified for mortality, stroke, myocardial infarction, bleeding, and need for reintervention for bleeding, if specified, and only in one series was a significant difference observed for mispositioning or a major paravalvular leak.

**Table 3.** **Postoperative outcomes in the comparative series**.

Author *et al*. [reference]	Valve	Mort.	Stroke	AMI	Bleed.	Reint.	PPMI	PVL > mild	mTVG	LOS
Aljalloud *et al*. 2023 [18]	P	5.5	-	-	6.7	-	8.4	1.7	12.1 (10.0–17.0)***	17.0 (13.0–27.0)***
	CS	2.4	-	-	10.8	-	10.8	0.0	9.0 (7.0–13.0)	13.0 (9.0–17.0)
Fischlein *et al*. 2021 [19]	P	1.0	1.5	1.0	4.4	1.0	10.6*	-	-	-
	CS	1.0	1.9	1.5	6.3	0.0	3.2	-	-	-
Fischlein *et al*. 2022 [20]	P	1.0	0.5	-	2.6	0.5	10.5*	0.5	-	13.3 ± 1.6
	CS	0.5	2.7	-	3.8	0.0	1.1	0.0	-	13.5 ± 1.5
	P	0.0	2.1	-	5.3	0.0	12.8*	0.0	-	12.6 ± 1.9
	CS	0.9	0.9	-	5.6	0.0	7.4	0.0	-	15.1 ± 1.9
Hernandez-Vaquero *et al*. 2020 [21]	P	6.4	-	7.8	-	-	10.7**	4.3***	13 (fig)	-
	CS	5.9	-	4.3	-	-	2.0	1.9	11 (fig)	-
Jayet *et al*. 2024 [22]	P ^S^	2.0	1.0	-	-	4.0	10.0	0.0	-	-
	CS	2.0	1.9	-	-	7.5	6.0	0.0	-	-
Kaitovic *et al*. 2023 [23]	P	2.0	1.0	-	-	8.9	3.0	-	-	-
	CS	0.4	1.3	-	-	8.7	1.7	-	-	-
Kapadia *et al*. 2024 [24]	P	2.0	0.0 ^B^	-	-	3.9	5.9	2.0	12–17^#^	-
	CS	2.0	2.9	-	-	5.9	7.8	3.0	10–12	-
Lorusso *et al*. 2021 [25]	P	-	1.5	-	4.4	-	-	-	-	-
	CS	-	1.9	-	5.6	-	-	-	-	-
Lorusso *et al*. 2022 [26]	P	-	-	-	-	-	10.4*	-	-	-
	CS	-	-	-	-	-	3.1	-	-	-
Paparella *et al*. 2021 [27]	P	0.7^B^	0.5	-	-	3.0	3.6	-	-	-
	CS	2.1	0.2	-	-	2.8	4.8	-	-	-
Robich *et al*. 2023 [28]	P	0.5	2.7	-	-	-	10.0	-	-	6.7 ± 0.3
	CS	0.9	2.1	-	-	-	6.8	-	-	7.7 ± 0.3
Santarpino *et al*. 2022 [29]	P	1.0	1.4	-	-	-	2.0	-	-	11
	CS	2.4	0.5	-	-	-	1.0	-	-	11
White *et al*. 2022 [30]	P, P ^S^	2.4	1.0	-	-	-	6.8**	-	6.9 ± 4.1	9.8 ± 10.0*
	CS	2.3	1.0	-	-	-	2.3	-	6.4 ± 1.3	8.9 ± 8.4
Zientara *et al*. 2024 [31]	P	0.0	-	-	-	5.0	-	-	-	8 (6–10)
	CS	0.5	-	-	-	4.1	-	-	-	8 (6–10)

AMI, acute myocardial infarction; bleed., bleeding; fig, data derived from figures in the included manuscript; LOS, length of stay; mort., mortality; mTVG, mean transvalvular gradient; PPMI, permanent pacemaker implantation; PVL, paravalvular leak; P, Perceval; CS, conventionally sutured; P ^S^, Perceval S; reint., reintervention; ^#^, depending on the size of the valve; *, *p* < 0.05; **, *p* < 0.01; ***, *p* < 0.001; ^B^, borderline.

**Fig. 6.**
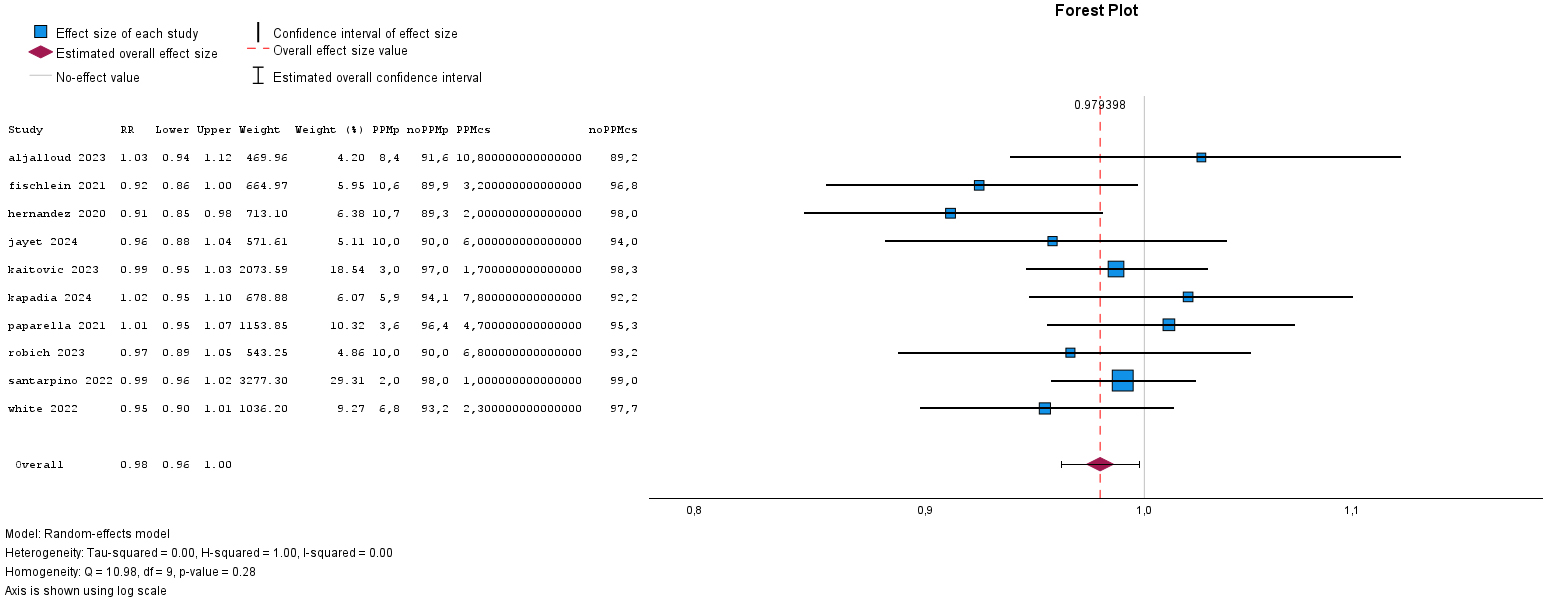
**Forest plot for postoperative permanent pacemaker implant**.

**Fig. 7.**
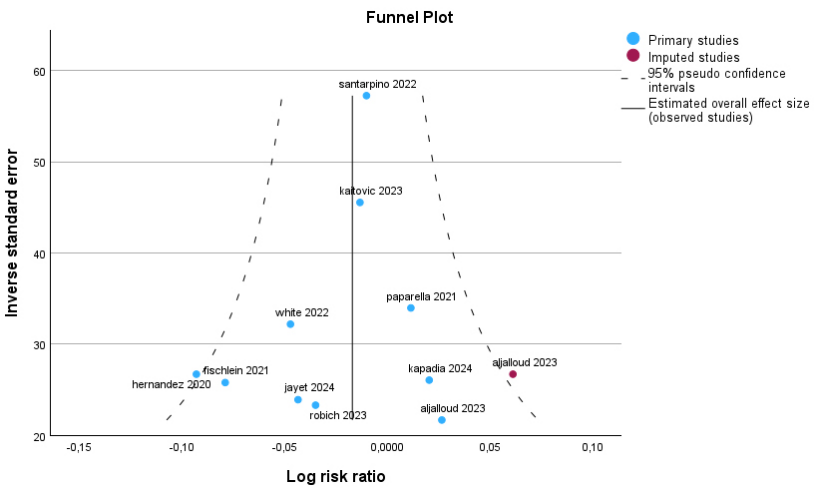
**Funnel plot for postoperative permanent pacemaker implant**.

Due to low rates and the absence of significant differences, these clinical adverse events were not included in the meta-analysis, except for the need for postoperative PPM implant following the Perceval valve. An increase was observed in several series [19, 20, 21, 26, 30], while in some series, the PPM implant was somewhat lower for the Perceval valve [18, 24, 27]. This result was confirmed by the meta-analysis (Figs. 6,7): A borderline significant difference was detected, with a value of 0.65 (0.41–1.02). The I^2^ was low, indicating no heterogeneity. The funnel plots showed no major publication bias. The size of the valve was the main predictor of the need for a PPM implant, while the absence of preoperative conduction defects had a significant protective effect [26]. A significant increase in length of stay could only be documented in one series [18]. Five manuscripts addressed postoperative thrombocyte counts [18, 22, 25, 27, 31]. Although a significantly lower number of thrombocytes was observed after the implantation of a Perceval valve, compared to conventionally sutured valves, there was no observed increase in bleeding events or need for transfusion.

#### 2.2.4 Meta-Analysis for the Need of Postoperative Permanent Pacemaker Implant

The metanalysis included 1841 patients for the Perceval valve and 3028 patients for the conventionally sutured valves. The inhomogeneity (I^2^) was low and almost all studies were within the funnel plot, indicating no publication bias.

Few results for long-term outcomes from these series were recorded. Some reports were restricted to the 1-year mortality rate, which was at a level below 4%. However, these data were compiled from the same RCT [19, 20]. The 4-year mortality rate for the Perceval valve was lower than that for conventionally sutured valves (2.9% versus 4.9%), but this difference was not statistically significant [24]. The 5-year survival rate showed similar mortality rates: 7.4% after implantation of a Perceval valve versus 12.6% for a conventionally sutured valve. This was also not statistically significant [31]. In one series, the transvalvular gradients seemed to remain stable over time, with low rates of paravalvular leaks [24].

Another 30 single-arm manuscripts (Tables 4,5; Ref. [32, 33, 34, 35, 36, 37, 38, 39, 40, 41, 42, 43, 44, 45, 46, 47, 48, 49, 50, 51, 52, 53, 54, 55, 56, 57, 58, 59, 60, 61]) were retained for analysis [32, 33, 34, 35, 36, 37, 38, 39, 40, 41, 42, 43, 44, 45, 46, 47, 48, 49, 50, 51, 52, 53, 54, 55, 56, 57, 58, 59, 60, 61], in which a large variation in sample size was observed, from large [33, 38, 51, 58], to medium-sized [43, 45, 48, 53, 54, 55, 59, 60] and smaller patient series [32, 34, 35, 36, 37, 39, 40, 41, 44, 46, 47, 49, 52]. The series, comparing the Perceval device with an Intuity valve, was treated as a single-arm series, because the data concerning the Perceval valve were presented separately [35]. In another series, a comparison was performed between isolated SAVR and SAVR combined with other procedures [42, 56, 61]. The data in Table 4 are presented as %, as mean ± standard deviation, or as median (interquartile range).

**Table 4.** **The preoperative and operative data from the single-arm series**.

Author *et al*. [reference]		N	Age (y)	ES-II (%)	Full sternot.	Part sternot.	RAMT	Conc. proced.	ACC (min)	CPB (min)
Brookes *et al*. 2021 [32]		130	74.4 ± 6.6	-	-	-	-	0	58.8 ± 19.4	81.3 ± 23.1
								100	69.5 ± 36.2	91.0 ± 44.5
Concistré *et al*. 2023 [33]		1652	73.5 ± 7.0	4.1 ± 6.3	54.4	25.4	19.6	35.9	61.0 ± 29.9	90.3 ± 42.2
Dokollari *et al*. 2023 [34]		101	71.2 ± 7.6	3.5 ± 4.5	100	0	0	0	47.3 ± 21.3	65.0 ± 29.6
D’Onofrio *et al*. 2020 # [35]		349	79.3 ± 6.4	4.4 ± 4.5	72.6	27.7	0	38.7	52 ± 14	71 ± 15
									59 ± 16	82 ± 22
Fabre *et al*. 2022 [36]		75 (E)	78.9 ± 5.5	2.6 ± 1.7	53.3	8.0	38.7	12.0	50.0 ± 16.6	72.3 ± 23.6
		211 (L)	76.2 ± 4.4	2.2 ± 1.6	36.0	38.4	35.6	24.2	56.8 ± 17.5	78.1 ± 23.2
Ferreira *et al*. 2022 [37]		196	77.2 ± 5.1	2.9 ± 2.2	-	-	-	>62	33.3 ± 14.1	45.6 ± 19.0
Fischlein *et al*. 2022 [38]		658	78.3 ± 5.6	10.2 ± 7.8	66.7	33.3	0	31.5	40.1 ± 18.1	64.8 ± 25.2
Hong *et al*. 2024 [39]		113	75.3 ± 8.4	STS 9.7 (4.4–18.0)	34.5	23.9	-	0	35.2 ± 3.9	82.7 ± 9.5
								100	77.6 ± 31.7	119 ± 40.5
Kim *et al*. 2021 [40]		121	74.7	3.0 ± 4.3	-	-	-	45.5	57.4 ± 37.9	91.4 ± 42.8
Lam *et al*. 2021 # [41]										
	No LBBB	135	72.6 ± 6.9	Log ES 6.4	-	-	-	38.5	48 (38–63)	75 (54–95)
	LBBB	39	75.2 ± 5.8	Log ES 8.0	-	-	-	61.5	61 (44–82)	98 (72–114)
Lamberigts *et al*. 2024 [42]		349	79.4 ± 5.5	2.9 (1.9–4.4)	47.6	47.9	4.6	0	38 (32–45)	61 (51–73)
		239	79.0 ± 4.7	5.7 (3.7–9.1)	86.6	13.4	0	100 CABG	70 (48–90)	108 (79–128)
		196	76.5 ± 6.9	6.4 (3.9–10.2)	85.7	14.3	9	100 MVR	89 (59–106)	118 (91–153)
Martínez-Comendador *et al*. 2021 # [43]		214	79 ± 5	2.7 ± 2.2	30.8	69.2	0	15.4	28 ± 14	40 ± 24
Mashhour *et al*. 2020 [44]		128	72.2 ± 8.5	8.2 ± 12.2	68.7	31.3	0	0		67 ± 22
								100		86 ± 34
Micovic *et al*. 2024 ## [45]		328	71.9 ± 6.4	2.9 ± 3.9	55.8	22.0	22.2	59.2	61.0 ± 28.8	92.9 ± 44.2
Müller *et al*. 2024 ## [46]		100	72.5 (67.3–79.0)	2.8 (1.7–5.4)	71	29	0	93	43 (34–59)	99 (78–137)
Nakayama *et al*. 2024 [47]		121	77 (74–80)	2.0 (1.5–3.3)	66.1	14.0	19.8	37.2	59 (51–77)	100 (74–114)
Niinami *et al*. 2023 [48]		204	77.5 ± 5.3	4.3 ± 5.7	74.5	0.5	25.0	46.6	68.0 (54.0–96.5)	108.0 (82.8–147.3)
Okiljevic *et al*. 2024 # [49]		100	73.2 ± 5.2	1.9 ± 2.1	0	100	0	0	46.6 ± 13.5	71.5 ± 25.5
		74	70.7 ± 5.7	1.5 ± 1.5	0	0	100	0	55.1 ± 14.4	83 ± 28
Pollari *et al*. 2023 [50]		547	76.4 ± 5.2	3.4 ± 2.6	29.6	68.7	1.6	32	53.4 ± 22.0	83.6 ± 28.0
Pollari *et al*. 2023 [51]		1934 (E)	77.6 ± 6.3	3.2 (1.8–6.4)	53.3	38.7	7.7	33.7	44 (32–60)	77 (53–90)
		670 (L)	75.8 ± 6.2	2.2 (1.3–3.7)	32.6	29.3	38.1	24.2	43 (32–62)	61 (47–84)
Ramsaransing *et al*. 2020 # [52]		110	74.2 ± 6.1	1.5 ± 0.9	0	100	0	0	54.0 ± 13.7	77.8 ± 21.2
Schizas *et al*. 2024 # [53]		205	76.4 ± 6.1	4.1 (0.9–14.1)	20.5	79.5	0	30.7	68.2 ± 51.5	108.3 ± 63.5
Sef *et al*. 2021 [54]		203	76.0 ± 6.2	1.9 ± 1.3	-	-	-	0	35 (24–76)	60.5 (39–153)
Solinas *et al*. 2020 [55]		503	78 ± 4	5.9 ± 8.4	-	-	100	0	50.3 ± 24.5	81.6 ± 30.8
								100	85.2 ± 28.9	129.6 ± 44.8
Szecel *et al*. 2021 [56]		201	79 ± 5	5.1 ± 5.5	70 $		Rare	0	39 ± 13	66 ± 22
		267	(all pts)	(all pts)	0			100	79 ± 32	116 ± 40
Szecel *et al*. 2022 # [57]		438 (E)	78.8 ± 5.1	6.4 ± 6.6	71			54.3	61.7 ± 31.7	94.9 ± 42.4
		246 (L)	78.2 ± 6.6	6.2 ± 7.0	66.5			56.9	60.6 ± 35.5	90.8 ± 47.0
Verlinden *et al*. 2022 # [58]		908	79.7 ± 5.2	4.1 (2.0–6.0)	64.3	33.8	1.9	44.9	60.5 ± 30.2	85.1 ± 40.9
		305 (E)								
		494 (L)								
Vilalta *et al*. 2022 [59]		76	78.2 ± 5.1	2.0 (1.4–3.2)	75.0	25.0	0	0	45.5 (39–62)	70 (58.5–88)
		253	77.4 ± 6.5	2.1 (1.3–3.7)	72.3	27.3	0	0	47 (38–68)	69 (55–95)
Vilalta *et al*. 2022 [60]		34	79.4 ± 4.4	1.9 (1.3–2.6)	68.6			27.8	45 (37–60)	69 (54–80)
		364	77.0 ± 6.4	2.0 (1.3–2.5)	64.5			22.2	44 (38–58)	64 (55–82)
Zubarevich *et al*. 2023 # [61]		85	66.6 ± 7.5	2.4 (1.9–5.0)	61.2 $			0	39.4 ± 17.8	63.5 ± 25.3
		75	71.2 ± 7.3	2.8 (2.7–4.1)	100	0	0	100 CABG	59.5 ± 19.8	86.9 ± 29.0
		40	71.5 ± 9.3	7.2 (5.7–16.0)	100	0	0	100 MVR	82.0 ± 35.5	112.8 ± 39.0

ACC, aortic cross-clamp time; all pts, all patients grouped; CPB, cardiopulmonary bypass time; Conc. proced., concomitant procedure; E, early era; ES, Euroscore; L, later era; LBBB, left bundle branch block; CABG, coronary artery bypass grafting; MVR, multiple valve replacement; N, number; sternot., sternotomy; STS, Society of Thoracic Surgeons; RAMT, right anterior mini-thoracotomy; $, numbers do not match the total provided in the included manuscript; #, Perceval S; ##, Perceval plus.

**Table 5.** **The short-term postoperative data from the single-arm series**.

Author *et al*. [reference]		Mort.	Stroke	Ac. inf.	PVL > mild	PPM	mTVG	pTVG	Reint.	Bleed.
Brookes *et al*. 2021 [32]		-	-	-	-	14.6	-	-	-	-
Concistré *et al*. 2023 [33]		0.8	0.4	0.2	0.2	5.7	13.3 ± 5.2	24.7 ± 10.5	0.7	1.3
Dokollari *et al*. 2023 [34]		2.0	3.0	-	2.0	8.0	14.7 ± 4.0	26.5 ± 7.0	-	-
D’Onofrio *et al*. 2020 # [35]		0.0	2.6	-	0.9	6.0	11.8 ± 4.7	22.5 ± 8.1	-	-
Fabre *et al*. 2022 [36]		5.3	-	-	-	16.0 (E)	14.3 ± 5.4	-	-	-
		4.2	-	-	-	5.6 (L)	12.5 ± 3.8	-	-	-
Ferreira *et al*. 2022 [37]		2.0	0.0	-	0.0	12.8	7.8 ± 3.6	-	-	-
Fischlein *et al*. 2022 [38]		3.7	2.4	-	0.8	8.2	10.3 ± 4.5	19.4 ± 8.0	1.0	-
Hong *et al*. 2024 [39]		2.6	0.0	-	0.0	2.6	10.5 ± 1.1	-	-	0.0
Kim *et al*. 2021 [40]		1.7	1.7	-	0.0	7.4	13.1 ± 3.8	24.9 ± 7.3	2.5	2.5
Lam *et al*. 2021 # [41]										
	No LBBB	0.0	2.2	0.7	-		-	-	5.2	-
	LBBB	2.6	5.1	0.0	-	5.4	-	-	7.7	-
Lamberigts *et al*. 2024 [42]		1.4	2.0	-	0.4	9.2	15 (11–18)	25 (20–32)	-	-
		5.0	1.7	-	(all pts)	9.2	(all pts)	(all pts)	-	-
		4.5	2.6	-		7.7			-	-
Martínez-Comendador *et al*. 2021 # [43]		0.5	0.9	-	-	9.8	-	-	1.9	-
Mashhour *et al*. 2020 [44]		2.3	1.6	-	0.0	3.1	9.9 ± 3.4	18.2 ± 5.8	5.5	-
Micovic *et al*. 2024 ## [45]		1.8	1.5	0.3	0.5	4.0	10.1 ± 4.7	18.7 ± 9.1	0.0	1.8
Müller *et al*. 2024 ## [46]		5.0	7.0	-	3.0	8.0	13 (10–18)	22 (18–29)	5.0	-
Nakayama *et al*. 2024 [47]		0.8	0.8	-	0.0	0.8	-	-	0.0	-
Niinami *et al*. 2023 [48]		0.5	-	-	-	4.4	13 (est)	-	0.5	-
Okiljevic *et al*. 2024 # [49]		2.0	1.0	-	1.0	2.0	-	-	5.0	-
		1.4	2.7	-	1.7	6.8	-	-	1.7	-
Pollari *et al*. 2023 [50]		3.3	3.3	0.4		8.7	15	29	-	-
Pollari *et al*. 2023 [51]		2.5	3.2	-		10.8 (E)	14.2 ± 5.8	26.5 ± 10.4	4.2	-
		1.4	3.1	-		6.3 (L)	14.2 ± 5.2	25.7 ± 9.3	5.1	-
Ramsaransing *et al*. 2020 # [52]		0.9	0.9	-	3.6		13.3	-	2.7	0.9
Schizas *et al*. 2024 # [53]		-	-	-		6.8	-	-	0.5	-
Sef *et al*. 2021 [54]		1.0	1.0	1.5	0.0		14.6 ± 8.8	24.6 ± 8.0	-	2.0
Solinas *et al*. 2020 [55]		0.8	2.2	-	-	5.2	11.9 ± 4.3	-	0.2	-
Szecel *et al*. 2021 [56]		3.2	1.8	-	-	7.9	-	-	2.3	-
Szecel *et al*. 2022 # [57]		3.9	1.8	-	1.5	11.0 (E)	15.5 ± 6.0	28.4 ± 10.3	3.0	-
		2.9	1.7	-	1.6	6.1 (L)	13.6 ± 5.3	24.4 ± 9.2	2.0	-
Verlinden *et al*. 2022 # [58]		3.9	1.5	-	0.0	12.5 (E)*	-	-	2.9	-
						7.7 (L)				
Vilalta *et al*. 2022 [59]		5.3	2.6	-	-	11.3	-	-	-	-
		5.1	3.1	-	-	6.5	-	-	-	-
Vilalta *et al*. 2022 [60]		-	-	-	-	-	-	-	-	-
Zubarevich *et al*. 2023 # [61]		2.4	0.0	-	0.0	3.5	6.2 ± 1.9	-	-	-
		4.0	1.2	-	0.0	1.3	5.9 ± 2.0	-	-	-
		20.0	0.0	-	0.0	10.0	5.3 ± 2.9	-	-	-

Ac. inf., acute myocardial infarction; bleed., bleeding; E, early era; est, estimated; L, later era; mort., mortality; mTVG, mean transvalvular gradient; PPM, permanent pacemaker; PVL, paravalvular leak; Reint., reintervention; pTVG, peak transvalvular gradient; LBBB, left bundle branch block; *, *p* < 0.05; #, Perceval S; ##, Perceval plus.

#### 2.2.5 Single-Arm Series Results

The mean patient age in these single-arm series was between 70 and 80 years, with a mean Euroscore mostly below 4%. The use of partial sternotomy as an approach is associated with prolonged ACC and CPB times, typically 5 to 10 minutes [35, 47, 49, 56]. However, the need for an associated procedure increased surgical times to a higher degree [35, 39]. In another series, in which an early era was compared with a later inclusion era, the need for a second cross-clamp was reduced from 1.3% to 0.9%; however, this reduction was not significant [36].

Mortality was low in several series following overestimations by the Euroscore II [33, 35, 41, 43, 47] or the Society of Thoracic Surgeon (STS) scores [48]. In one series, the observed mortality rate of 20% was very high for patients undergoing multiple valve replacements, and this outcome was underestimated by a Euroscore of 7.2% [61], whereas in another series, the discrepancy (6.4% versus 4.5%) was much lower [42]. The rates for myocardial infarction and stroke were also low, except for one series [46]. Reintervention for valve migration or paravalvular leak was absent [18, 52] or occurred at a rate of no more than 1% [46, 53, 61]. This reintervention rate was somewhat higher for patients who underwent upper partial sternotomy, but was absent in patients with RAMT [49]. As in the comparative series, the reintervention rate for bleeding was higher, at 2–5% [46, 49, 58]; acute tamponade was another reason for reintervention [52, 58]. Paravalvular leaks and mispositioning of the Perceval valve were much less of an indication for reintervention, compared to postoperative bleeding. One series compared upper partial sternotomy with RAMT as an approach; RAMT appeared to yield superior results compared to the output of chest tubes [49].

In one series, postoperative left bundle branch block (LBBB) was the most commonly observed conduction defect and persisted in approximately two-thirds of the patients during hospitalization. The occurrence of LBBB increased the need for PPM significantly during hospital stay and at follow-up. A prior right bundle branch block (RBBB) was the strongest predictor of the need for a PPM, with an odds ratio of almost 3. Three-year survival was not affected by PPM implantation. In an important portion of patients, atrioventricular conduction recovered during follow-up [58, 59, 60]. The need for postoperative conduction defects and the need for PPM implant were remarkably low in some series [47], while in most series, the rate of PPM implantation was above 5%. The need for PPM was high in multivalve procedures [61]. One series reported a recovery rate of conduction in 7 out of 10 patients who underwent PPM implantation [41]. The effect of improving surgical expertise over time on the rate of postoperative PPM implantation was observed in four series [36, 51, 57, 58]. This effect was also documented with respect to gradients across the device and more than mild central valvular leaks [57]. These improvements could relate to patient selection, surgical technique refinement, and more accurate sizing.

#### 2.2.6 Long-Term Outcomes

Nine series are available with a long-term outcome of at least 5 years, which seems to be the minimum follow-up duration needed to derive valid conclusions. Some series reported a favorable outcome with a survival rate of over 95% at 5 years, which is remarkably high for a patient series with a mean age of 75 years or older [39, 55]. Other series reported a 7-year survival rate of 87.9%. Diastolic dysfunction or female gender were predictors for long-term mortality [34]. In other series, the 5-year mortality rate was higher, at 29% for a patient group with a mean age of 77 years [37] and 27.3% for a patient group with a mean age of 78 years [38]. A lower 5-year survival rate of 64.8% was observed in a third series [53]. The need for additional procedures to SAVR could be an attributing factor for the observed increase in mortality [46].

The freedom from reoperation of 99.2% at almost 5 years was high [33, 55], and a need for reoperation was not observed in some series [39]. The rate of prosthetic valve endocarditis (PVE) was low, ranging from 0.6% to 1% at a follow-up of at least 5 years [34, 55], while this complication was not reported in other series [37, 39]. The longest follow-up, at 8 years, recorded a PVE rate of 2% [50]. Structural valve degeneration (SVD) at 5 years was also low (0.8%), and this condition was mostly treated using TAVI [55]. The median freedom from SVD was just over 10 years, with increased age noted as a significant protective factor [50]. In some series, no cases of SVD were observed [37]. The hemodynamic performance of the Perceval valve remained stable during long-term follow-up [33, 38, 39, 55], which may explain the adequate reduction in left ventricular mass index (LVMI) [38, 39, 55]. The development of paravalvular leaks also remained below 1% [38, 50], although there was a rise in central valvular leaks in one series, which was without clinical consequence [38].

## 3. Discussion 

Following the introduction of TAVI, the treatment of symptomatic aortic valve disease has undergone profound changes. The place of SAVR in mid-risk older patients is a matter for debate in the polarization between SAVR and TAVI. Both treatment modalities have evolved through the implementation of new techniques and new devices. The Perceval device is an attempt to integrate the “best of both worlds” by combining the excision of the diseased valve with decalcification of the annulus, followed by device deployment without the need for suturing. The self-anchoring properties of the Perceval device facilitate its rapid deployment under direct vision, minimize manipulation of the aortic root, and shorten surgical times [58, 60].

The implantation of the device requires attention to certain technical details to avoid mispositioning, oversizing, and damage to the conduction system. These details include adequate decalcification of the annulus, precise placement of the three guiding sutures below the annulus, correct sizing, avoiding excessive traction on the suspension sutures during device deployment, and reducing the extent and duration (or even omitting) of balloon inflation after deployment. Moreover, avoiding oversizing would prevent a pinwheel effect on the leaflets, potentially resulting in a higher transvalvular gradient [58].

The use of the Perceval device results in shortened deployment times and, thus, reduced ACC and CPB times in all included series [18, 22, 23, 24, 25, 27, 28, 30, 32, 34, 35]. This was observed for both full and partial sternotomy [20]. These operative times could theoretically be considered predictors of mortality [34, 35], which would make this technique more suitable for older, high-risk patients with multiple comorbid conditions [18]. Nevertheless, in one series, the faster implantation had no impact on the 30-day mortality [18]. This lack of effect might be due to the nonlinear relationship between CPB time and complication rates. The potential benefit of a shorter bypass time with sutureless valves may be most relevant in patients requiring long procedural times for complex, multiple procedures or redo procedures [31, 34].

The introduction of minimally invasive SAVR (MI-AVR), such as partial sternotomy and RAMT, appears to have been facilitated by the Perceval devices, especially in patients requiring isolated SAVR [53, 58], without making the implantation itself more difficult [18]. However, a wide variation in the use of MI-AVR was observed among the included series, depending on the choice and expertise of the surgeon [31]. The use of MI-AVR increased surgical times to a limited degree [20], lowered the need for transfusion [27, 28], and reduced postoperative morbidity and mortality [24, 26, 55] in several series—the hemodynamic outcome after MI-AVR was comparable [20]. Meanwhile, repositioning of the device, if necessary, could be conducted using MI-AVR [18].

The 30-day mortality rate in the included series is generally low, and in some cases, it is lower than the mortality rate predicted by the already low Euroscore II [20, 58]. In other series, the Euroscore II predicted the observed mortality fairly well [22, 23, 24] or showed a limited overestimation of mortality [27, 29]. One series with a higher Euroscore II of 5.4% predicted the observed 30-day mortality of 5.9% fairly well [21]. Sutureless valves showed equivalent results compared to sutured valves with respect to cerebrovascular and cardiovascular postoperative events at 30 days and one year [19]. The mean and peak transvalvular gradients were higher in the Perceval valve than in the conventionally sutured valves [18, 21]. This may be due to the lower radial force of the device leading to stent deformation, flutter, and reduced mobility of the cusps [62]. This higher gradient was observed across all device sizes [21, 24]. One subset in an RCT showed no significant differences in gradients across both valve types, nor reduction in LVMI at the one-year follow-up [63].

Migration, dislocation, and severe paravalvular leak (PVL) needing reoperation were rarely observed in the included series. In most comparative groups, no significant difference was documented with respect to more than a mild PVL [18, 20, 22]. If a PVL was present, this difference disappeared over time [24]. Notably, a more than mild PVL was significantly higher in only one comparative series in patients administered Perceval devices compared to conventionally sutured valves; however, the percentage remained low [18]. Meanwhile, the rate of PVL reached or exceeded 3% in only two single-arm series [46, 52]. A PVL is often caused by incorrect valve sizing, highlighting the importance of technical details when using these new valve technologies. The Perceval valve provides an optimal sealing of the aortic anulus, demonstrating the reliability of its technology. Reoperation for bleeding or tamponade was also low and comparable between the valves under study [19, 20, 27]. The rate of reintervention was higher in some series, but remained similar for both valves [22, 23, 24, 31].

Thrombocytopenia was one of the immediate postoperative complications, and many concerns have been raised about the potential early complications. Typically, the nadir of the platelet count was at the second [31] or third postoperative day for both valves [22, 25]. This nadir was deeper for Perceval valves compared to conventionally sutured valves. Except for platelet transfusion, no additional blood products or re-exploration for bleeding were needed [40]; the mechanisms underlying low platelet counts are not fully understood. A higher mean platelet volume and platelet distribution width after implantation with a Perceval valve could indicate platelet activation, but without major complications. Low platelet count could also be a consequence of stent deformation and flutter [62]. Meanwhile, an increased consumption of platelets associated with hemolytic or inflammatory processes is unlikely without a corresponding difference in lactate dehydrogenase, C-reactive protein, or white blood cell (WBC) count. Apoptosis of platelets induced by activating N-methyl-D-aspartate receptors and membrane rupture by the nitinol frame have been suggested, as possible mechanism but this remains speculative [22, 31]. The role of the postoperative higher aortic transvalvular gradients in the Perceval group also seemed unlikely [22]. In the sutured valve population, age and CPB time were predictive of a low platelet count, whereas no predictors were identified in the Perceval group [31]. In one small series, postoperative thrombocytopenia was predicted by ages above 80 years, a Euroscore above 2.9%, a low preoperative platelet count, and the presence of dialysis. Low postoperative thrombocyte levels were less commonly observed in patients who underwent Perceval implantation through minimally invasive surgery [47].

A new-onset LBBB occurred in over one-third of the patients and persisted in about three-quarters of these cases. Oversizing and reliance on radial force were risk factors affecting the stability of the valve. The occurrence of this conduction defect did not affect 30-day mortality; however, the need for a PPM implant during follow-up was significantly increased in these patients. The occurrence of new-onset LBBB was associated with a decrease in left ventricular ejection fraction (LVEF), but not with an increase in the rate of readmission for heart failure or mortality during long-term follow-up [59, 60]. The need for postoperative PPM implant was approximately 10% in many comparative series and similar to the requirement following TAVI; however, the rate was higher compared to conventionally sutured valves. This observation did not depend on the type of surgical access or the need for concomitant procedures [20, 26]. The mean time for PPM implantation was just over a week after SAVR [32, 58, 60]. A preoperative RBBB was an important predictor; however, there was also an association with a longer PR interval, older age, and the need for repositioning of the device. The need for a PPM decreased over time, illustrating the importance of a learning curve: oversizing and high ballooning pressure were avoided in later eras [22, 34]. Lowering the height of the sealing collar, as in the Perceval S and Perceval PLUS, could contribute to the reduced need for a PPM [58, 60]. The need for a PPM was not observed if a conversion to a conventionally sutured valve was made after dislocation of the Perceval device [58].

Long-term outcomes, including survival, hospital readmission, repeat intervention, stroke, atrial fibrillation, and myocardial infarction, were comparable between Perceval devices and other valves [24, 34] or were more favorable for Perceval valves [20]. Age, Euroscore II, and postoperative stroke were predictive for long-term survival, but the use of a sutureless valve had no effect in this respect [23]. The valvular gradients remained stable at 1-year follow-up: In one series, the differences observed between Perceval and conventionally sutured valves disappeared over time. Consequently, the mid-term left ventricular (LV) dimensions and function seemed equally improved after implantation of both valves [24]. The 5-year durability of the Perceval device seems reasonable, with low or absent reoperation rates [28, 31]; the incidence of endocarditis was also low [20, 23, 34]. However, the limited follow-up duration warrants caution when interpreting these findings, and a longer observation period is necessary to reveal any potential complications.

The Perceval valve also exhibits several attractive features in the event of a future need for a valve-in-valve TAVI. The visibility of the frame during radioscopy is an advantage for determining the landing zone; however, care should be taken to pass the outflow ring through the inside during the TAVI procedure, since this frame is not always attached to the aortic wall. The Perceval device features a self-expandable nickel–titanium alloy stent, which allows for overexpansion of up to 2.5 mm during the procedure. This design also lowers the risk of coronary artery obstruction. A no-contrast TAVI procedure is possible, which is particularly important for patients with chronic renal dysfunction or contrast allergy. However, in patients with shallow roots and low coronary artery implantation, a TAVI procedure in a degenerated Perceval valve remains challenging. The valve-in-valve TAVI was safe and showed good clinical results with excellent hemodynamic performance [64]. These findings were confirmed in a recently published review article. Furthermore, the postprocedural PPM rate was lower compared to valve-in-valve TAVI in conventionally sutured valves, likely due to the flexibility of the Perceval device [65]. Conversely, in patients with small annuli, for whom valve-in-valve TAVI is not a viable option, redo-SAVR with a Perceval valve may be an alternative—this approach offers advantages in terms of ease of deployment and shorter surgical times. In patients needing redo-SAVR after a Bentall procedure, dissection of the root and reimplantation of coronary arteries can be avoided. Moreover, the long-term effects of valve-in-valve TAVI remain uncertain, with a potential for a less favorable outcome [66].

Another aspect is the comparison of the use of the Perceval valve and TAVI as an alternative, since both options have been developed for patients with a higher surgical risk. Two PSM analyses compared TAVI with SAVR using a Perceval device. In one series, there was a higher rate of PVL after TAVI, but with comparable need for PPM, mortality, and stroke rates [67]. The second PSM series compared transapical TAVI with a Perceval device. Patients undergoing TAVI had a higher rate of peripheral artery disease, which prohibited a transfemoral access. Of the surgical patients, 61% also received CABG, while a later PCI was anticipated in 75% of the TAVI patients. Moreover, there was an increased need for reintervention and blood transfusion in the surgical group [68]. However, with respect to these groups, the 56- and 59-patient pairs could be considered too small to observe differences in other postoperative complications or mortality. A meta-analysis comparing SAVR with a Perceval valve and TAVI was based on larger series, totaling 3764 patients for the comparative series. This analysis showed that surgical patients had a lower rate of vascular complications, early PVLs, conduction defects, and need for a PPM implant. Surgical patients had a higher rate of early atrial fibrillation and bleeding. The rate of other complications was low for both groups. The mean transvalvular gradient was 2.27 mmHg higher in the surgical group. This gradient increased from 10.5 mmHg to 11.2 mmHg over 5 years. The single-arm analysis for surgery also showed low 5-year rates for endocarditis, stroke, valve degeneration, and need for valve explantation, which aligns with the findings in the current analysis. Coupled with the advantages of shorter surgical times, the option of minimally invasive SAVR, as well as the possibility of valve-in-valve TAVI in cases of degeneration, makes the Perceval valve a competitive option in managing patients with aortic disease who fall into a “gray zone” [69].

### Limitations

This analysis is primarily based on a cumulative retrospective series, which presents inherent limitations. Due to the propensity score matching analysis in six series, this problem has been mitigated. The use of randomized trials also addressed this issue. Manuscripts describing the outcome of the Perceval valve in difficult situations, such as asymmetric bicuspid aortic valves, aortic valve regurgitation, and endocarditis, have not been included, despite reasonable results being reported.

## 4. Conclusions

There is a clear shortening in ACC and CPB times following implantation of the Perceval device. This could translate into improved clinical outcomes, particularly for patients with prolonged surgical times; however, this was not evident in the included series. The Perceval valve could serve as a so-called bridge between conventional SAVR and TAVI in older patients with an intermediate surgical risk. Present results have shown acceptable clinical and hemodynamic outcomes, which appear to be durable. The higher postoperative PPM implantation rate might improve following an adaptation of current surgical techniques. A minimally invasive approach for aortic valve replacement can be facilitated through the implantation of Perceval valves. The 10-year durability of this valve needs further investigation. Nonetheless, the advantages of using the Perceval valve could outweigh the drawbacks.

## Availability of Data and Materials

All data analyzed during this study were obtained from previously published articles, which are publicly available. The datasets used for the meta-analysis (e.g., extracted effect sizes, sample sizes) and the statistical code are available from the corresponding author upon reasonable request.

## Supplementary Material

Supplementary material associated with this article can be found, in the online version, at https://doi.org/10.31083/RCM39463.
